# Cynaroside Induces G1 Cell Cycle Arrest by Downregulating Cell Division Cycle 25A in Colorectal Cancer

**DOI:** 10.3390/molecules29071508

**Published:** 2024-03-28

**Authors:** Shan Lei, Wenpeng Cao, Zhirui Zeng, Lu Wang, Jinzhi Lan, Tengxiang Chen

**Affiliations:** 1 Department of Physiology, School of Basic Medical Sciences, Guizhou Medical University, Guiyang 550009, China; leishan@gmc.edu.cn (S.L.); zengzhirui@gmc.edu.cn (Z.Z.); wanglu@gmc.edu.cn (L.W.); lanjinzhi@gmc.edu.cn (J.L.); 2 Transformation Engineering Research Center of Chronic Disease Diagnosis and Treatment, Guizhou Medical University, Guiyang 550009, China; 3 Department of Anatomy, School of Basic Medical Sciences, Guizhou Medical University, Guiyang 550009, China; caowenpeng@gmc.edu.cn

**Keywords:** cynaroside, cell division cycle 25A, colorectal cancer, cell cycle, proliferation

## Abstract

Natural chemicals derived from herbal plants have recently been recognized as potentially useful treatment alternatives owing to their ability to target a wide range of important biological molecules. Cynaroside is one of these natural compounds with promising anticancer activity for numerous tumor types. Nevertheless, the anticancer effects and molecular mechanisms of action of cynaroside on colorectal cancer (CRC) remain unclear. In this study, cynaroside was found to markedly inhibit CRC cell proliferation and colony formation in vitro. Cynaroside also inhibited cell proliferation in vivo and decreased the expression of KI67, a cell nuclear antigen. RNA sequencing revealed 144 differentially expressed genes (DEGs) in HCT116 cells and 493 DEGs in RKO cells that were enriched in the cell cycle signaling pathway. Cell division cycle 25A (CDC25A), a DEG widely enriched in the cell cycle signaling pathway, is considered a key target of cynaroside in CRC cells. Cynaroside also inhibited DNA replication and arrested cells in the G1/S phase in vitro. The expression levels of CDC25A and related G1-phase proteins were significantly elevated after CDC25A overexpression in CRC cells, which partially reversed the inhibitory effect of cynaroside on CRC cell proliferation and G1/S-phase arrest. In summary, cynaroside may be used to treat CRC as it inhibits CDC25A expression.

## 1. Introduction

Colorectal cancer (CRC) is a prevalent malignancy of the digestive tract that is induced by the dysregulation of the proliferation, differentiation, and migration of colonic mucosal epithelial cells [1]. Following lung and breast cancer, CRC is the third most prevalent cancer globally and has the second highest cancer fatality rate [2]. Currently, the treatment methods for CRC include chemotherapy, radiotherapy, immunotherapy, targeted therapy, and traditional Chinese medicine (TCM) [3]. Surgical resection in conjunction with chemotherapy is the mainstream comprehensive approach for CRC treatment; however, these treatments have not reduced the morbidity, mortality, or recurrence rate of CRC in patients [4]. Hence, the design of more effective treatments is a priority. In recent years, TCM and its active extracts have played an important role in colorectal cancer treatments owing to its advantages, such as multiple pathways, multiple targets, and low toxicity and side effects [5]. Several TCM components have been demonstrated to have good inhibitory effects on the growth, metastasis, recurrence, and drug resistance of CRC [6,7].

Cynaroside, also known as luteolin-7-O-glucoside, is a flavonoid widely found in the medicinal plants of Rosinaceae. Cynaroside exerts anti-inflammatory, antiviral, anti-tumor, cardiovascular system, nervous system protection, and other biological activities [8,9]. Shao et al. revealed that cynaroside reduces the proliferation and promotes the apoptosis of cervical cancer cells by inhibiting the MAPK and mTOR signaling pathways [10]. According to Ji et al., extracted cynaroside has good anti-tumor activity and can effectively inhibit the proliferation of gastric cancer cells [11].

Cell division cycle 25A (CDC25A), a component of the CDC25 family, is a bi-specific protein phosphatase that hydrolyzes tyrosine and serine/threonine dephosphorylation [12]. CDC25A performs an integral function in the progression of the cell cycle and can promote G1/S- and G2/M-phase transformations by activating the cyclin–CDK complex, which is an important target of the DNA damage response [13]. CDC25A is an oncogene that is strongly associated with the onset and progression of tumors and is an effective therapeutic target for liver cancer, breast cancer, colon cancer, and other tumors [14,15,16]. 

The purpose of this study was to evaluate the role and key molecular processes of cynaroside in CRC and provide a theoretical basis for CRC treatment. Based on the results, cynaroside downregulated CDC25A, mediated G1/S-phase arrest, and suppressed CRC cell proliferation. Such findings revealed a new mechanism for the anti-CRC activity of cynaroside, which is expected to serve as a novel clinical treatment for CRC.

## 2. Results

### 2.1. Cynaroside Suppresses the Proliferation and Colony Formation of CRC Cells

**Figure 1:**
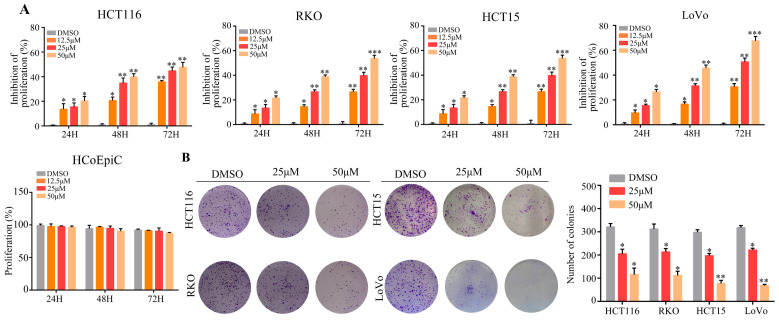
Cynaroside inhibits CRC cell proliferation in vitro. (**A**) HCT15, RKO, HCT116, LoVo, and HCoEpiC cells were treated with different concentrations (0, 12.5, 25, and 50 μM) of cynaroside, and the CCK-8 assay was used to detect the proliferation in each group. (**B**) The colony formation assay was used to detect the colony formation ability of CRC cells treated with different concentrations (0, 25, and 50 μM) of cynaroside. * represents *p* < 0.05; ** represents *p* < 0.01; *** represents *p* < 0.001. *n* = 3. The control group was used for comparison. Data are shown as mean ± SD.

The role of cynaroside in CRC cell growth in vitro was assessed here in HCT116, HCT15, RKO, LoVo, and the human colonic epithelial cell, HCoEpiC. Cells were treated with different doses of cynaroside (0, 12.5, 25, and 50 μM). Based on the CCK-8 assay results, cynaroside considerably attenuated the growth of CRC cells in contrast to the control at 24, 48, and 72 h, and cynaroside was not cytotoxic to HCoEpiC (Figure 1A). As 25 and 50 μM of cynaroside had a significant inhibitory effect on CRC cells, 25 and 50 μM were used for subsequent studies. After 10 days of culture, the colony formation rate of CRC cells implanted in six-well plates was calculated. According to the findings, cynaroside markedly reduced the colony formation rate of CRC cells compared to the control (Figure 1B). These results indicate that cynaroside inhibits cell growth in CRC cells.

### 2.2. Cynaroside Suppresses the Proliferative Potential of CRC Cells In Vivo

**Figure 2:**
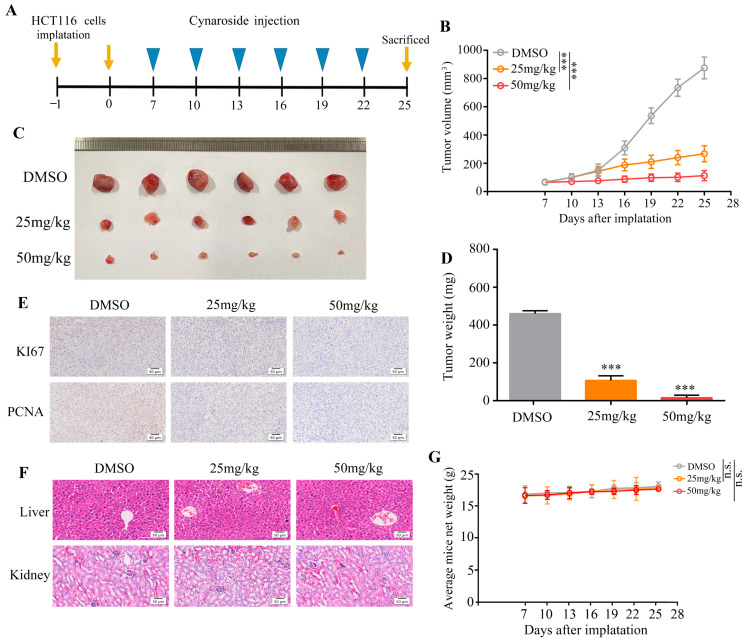
Cynaroside represses the proliferation rate of HCT116 cells in vivo. (**A**) Model diagram of the animal experiments. (**B**,**C**) The proliferation of tumor tissues treated with DMSO and cynaroside. (**D**) Weight of tumor tissues treated with DMSO and cynaroside. (**E**) The expression of PCNA and KI67 in the tumor tissues treated with DMSO and cynaroside. (**F**) HE staining was performed to determine injuries in the liver and kidney of mice treated with DMSO and cynaroside. (**G**) Body weight changes in mice treated with DMSO and cynaroside. *** represents *p* < 0.001, n.s. represents not significant, *p* < 0.001. *n* = 5. The control group was used for comparison. Data are shown as mean ± SD.

To evaluate the therapeutic potential of cynaroside in vivo, HCT116 cells were administered to nude BALB/C mice via a subcutaneous injection. The mice were randomly classified into two groups: the control group (DMSO) and cynaroside group (25 and 50 mg/kg). Tumor growth was rapid in the control group (DMSO); however, cynaroside significantly inhibited tumor growth (Figure 2A–C) and reduced tumor weight (Figure 2D). In addition, the protein expression levels of KI67 and PCNA were lower in tumor samples treated with cynaroside than those treated with DMSO (Figure 2E). HE staining revealed no liver or kidney injury in nude mice treated with cynaroside (Figure 2F). There was no change in body weight in nude mice treated with cynaroside (Figure 2G).Thus, cynaroside had an obvious anti-tumor effect in vivo and few toxic side effects.

### 2.3. Cynaroside Suppresses the Proliferative Potential of CRC Cells In Vivo

**Figure 3:**
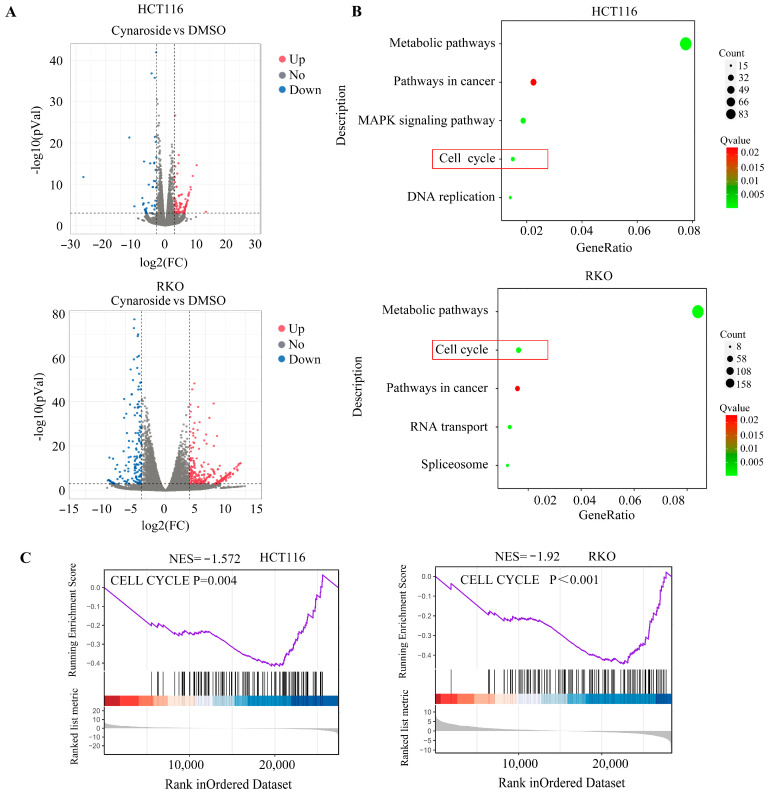
High-throughput sequencing analysis of HCT116 and RKO cells treated with DMSO and cynaroside. (**A**) Differentially expressed genes in CRC cells treated with DMSO and cynaroside. (**B**) KEGG analysis was performed to determine the pathways of the differentially expressed genes. (**C**) Gene enrichment plots showing the series of genes enriched in the cell cycle. *n* = 3. The control group was used for comparison.

To ascertain the molecular mechanisms of cynaroside in CRC cells, high-throughput sequencing was performed. A total of 43 downregulated genes and 101 upregulated genes with | logFC | ≥ 3 and an adjusted *p*-value of <0.001 were identified in HCT116 cells treated with cynaroside, and 154 downregulated genes and 339 upregulated genes were identified in RKO cells (Figure 3A). Through KEGG analysis, the DEGs induced by cynaroside treatment in HCT116 cells were found to be remarkably enriched in transcriptional abnormalities in metabolic pathways, DNA replication, pathways in cancer, MAPK signaling, and cell cycle pathways. The genes differentially expressed by cynaroside in RKO cells were significantly enriched in transcriptional abnormalities in metabolic pathways, the cell cycle, pathways in cancer, RNA transport, and spliceosomes (Figure 3B). Further, based on gene set enrichment analysis (GSEA), many cell cycle-related genes were enriched in the cynaroside group and were negatively associated with cynaroside treatment (Figure 3C).

### 2.4. CDC25A Is a Key Target of Cynaroside

**Figure 4:**
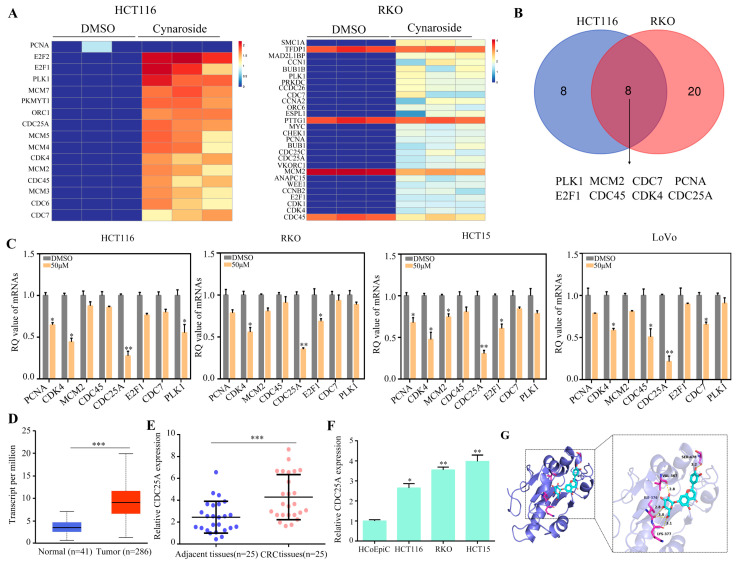
CDC25A is a key target of cynaroside. (**A**) Heatmap showing the changes in different genes involved in the cell cycle in HCT116 and RKO cells. (**B**) Schematic illustration showing the overlapping of differential genes enriched in the cell cycle based on RNA sequencing after the treatment of HCT116 and RKO cells with cynaroside. (**C**) qRT-PCR was used to detect the mRNA levels of PLK1, MCM2, CDC7, PCNA, E2F1, CDC45, CDC25A, and CDK4 in CRC cells treated with different concentrations (0, 25, and 50 μM) of cynaroside. (**D**) CDC25A expression in CRC tissues and normal tissues according to the data from TCGA. (**E**) qRT-PCR was used to detect the expression of CDC25A in CRC tissues and adjacent tissues. (**F**) qRT-PCR was used to detect the expression of CDC25A in CRC cell lines and the human colonic epithelial cell, HCoEpiC. (**G**) The binding mode of cynaroside with CDC25A and the 3D illustration of the details of the interaction. Purple represents the CDC25A protein; green represents cynaroside. * represents *p* < 0.05; ** represents *p* < 0.01; *** represents *p* < 0.001. *n* = 3. The control group was used for comparison. Data are shown as mean ± SD.

The differential genes enriched in cell cycle pathways in cynaroside-treated HCT116 and RKO cells were further analyzed using a heat map (Figure 4A). Based on the Venn diagram, the regulation of PLK1, MCM2, CDC7, PCNA, E2F1, CDC45, CDC25A, and CDK4 decreased continuously in HCT116 and RKO cells (Figure 4B). In addition, qRT-PCR revealed that the transcriptional levels of CDC25A, PCNA, CDK4, E2F1, and PLK1 were remarkably lower in CRC cells following treatment with cynaroside (Figure 4C). Among them, CDC25A had the most significant decrease after treatment with 25 μM of cynaroside. By using the TCGA database to compare the gene expression levels between CRC tissues (*n* = 286) and normal tissues (*n* = 41), the level of CDC25A expression was found to be considerably elevated in CRC tissues compared to normal tissues (Figure 4D). qRT-PCR also revealed that the level of CDC25A expression was elevated in CRC tissues compared to normal tissues (Figure 4E). Similarly, CDC25A expression levels were higher in the CRC cell lines, including HCT116, RKO, and HCT15, relative to the human colonic epithelial cell line, HCoEpiC (Figure 4F). Molecular docking technology was then used to analyze the binding model of cynaroside and the CDC25A protein. According to the 3D drawing, cynaroside binds to VAL367, ILE376, LYS377, and SER478 in the CDC25A protein in a stabilizing manner (Figure 4G). Collectively, these results illustrate that CDC25A is a critical target of cynaroside.

### 2.5. Cynaroside Suppresses CRC Cell DNA Replication and Induces CRC Cell G1/S-Phase Arrest In Vitro

**Figure 5:**
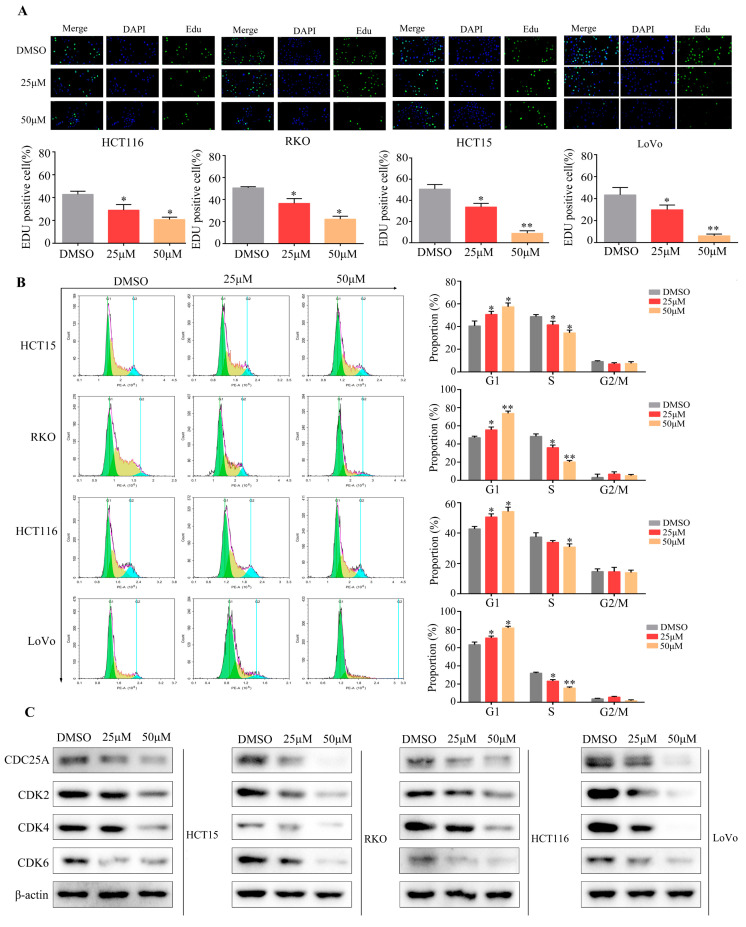
Cynaroside induces CRC cell arrest in the G1/S phase in vitro. (**A**) EDU assays were used to detect the EDU positive rate in CRC cells treated with DMSO and cynaroside. (**B**) CRC cells were treated with different concentrations (0, 25, and 50 μM) of cynaroside, and flow cytometry was used to detect the cell distribution in each group. (**C**) Western blot was used to detect the expression of CDK4, CDK6, and CDK2 in CRC cells treated with different concentrations (0, 25, and 50 μM) of cynaroside. * represents *p* < 0.05; ** represents *p* < 0.01. *n* = 3. The control group was used for comparison. Data are shown as mean ± SD.

We explored the impact of cynaroside on DNA replication and cell cycle processes in CRC cells. The Edu assay results revealed that the positive rate of Edu in CRC cells treated with cynaroside was lower than that in cells treated with DMSO (Figure 5A). Cynaroside markedly lowered the number of CRC cells in the S phase and remarkably elevated the percentage of CRC cells in the G1 phase, according to the flow cytometry findings (Figure 5B). Western blotting revealed that cynaroside markedly decreased the expression of biomarkers involved in the G1/S phase in CRC cells. These biomarkers included CDK4, CDK2, and CDK6 (Figure 5C). According to these findings, cynaroside markedly suppresses DNA replication in CRC cells and induces G1/S-phase arrest.

### 2.6. Overexpression of CDC25A Attenuates the Inhibitory Impacts of Cynaroside on the Proliferative Potential of CRC Cells

**Figure 6:**
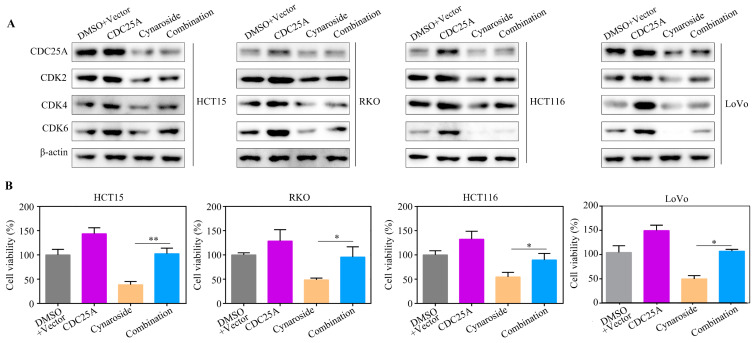
Overexpression of CDC25A reverses the inhibitory effects of cynaroside on the proliferation of CRC cells. CRC cells were treated with DMSO, cynaroside, CDC25A plasmid, and cynaroside + CDC25A plasmid, respectively. (**A**) Western blot was used to detect the expression of CDC25A, CDK4, CDK6, and CDK2 in each group of CRC cells. (**B**) CCK-8 was used to detect the proliferation rate of CRC cells in each group. * represents *p* < 0.05; ** represents *p* < 0.01. *n* = 3. The control group was used for comparison. Data are shown as mean ± SD.

**Figure 7:**
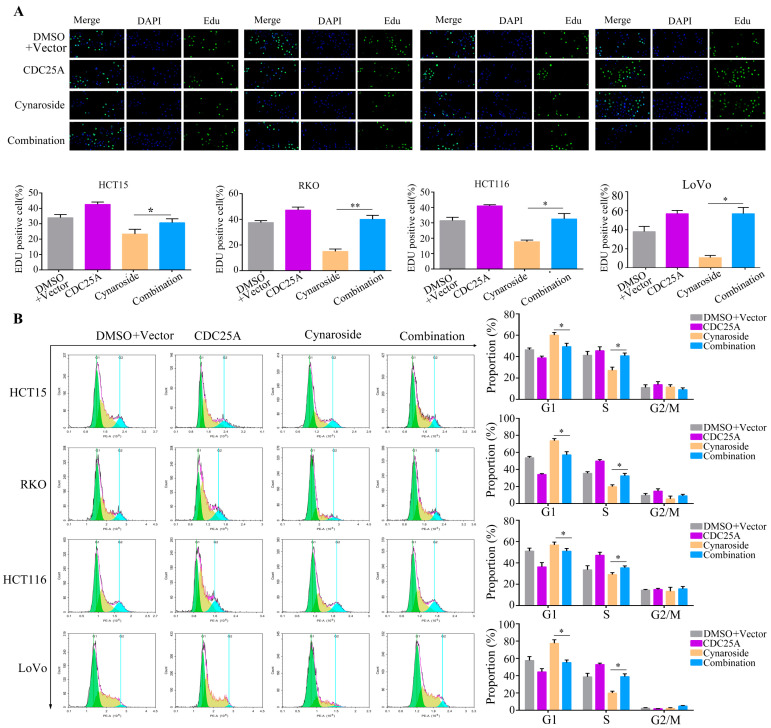
Overexpression of CDC25A reverses the G1/S-phase blocking effect of cynaroside on CRC cells. RC cells were treated with DMSO, cynaroside, CDC25A plasmid, or cynaroside + CDC25A plasmid. (**A**) EDU assays were performed to determine the DNA replication rate of CRC cells in each group. (**B**) Flow cytometry was performed to determine the distribution of CRC cells in each group. * represents *p* < 0.05; ** represents *p* < 0.01. *n* = 3. The control group was used for comparison. Data are shown as mean ± SD.

CDC25A has been demonstrated to be an oncogenic gene in several tumors; therefore, we hypothesized that CDC25A is involved in cynaroside-induced biological functions. Therefore, CDC25A-overexpressed plasmid was transfected into CRC cells prior to cynaroside treatment. Western blotting revealed that the expression levels of CDC25A, CDK4, CDK6, and CDK2 were considerably lower in CRC cells following cynaroside treatment. However, CDC25A, CDK4, CDK6, and CDK2 expression levels were partially increased in CRC cells after treatment with cynaroside and CDC25A overexpression (Figure 6A). The CCK-8 assay revealed that the inhibitory effect of cynaroside on the proliferative potential of CRC cells was alleviated after the overexpression of CDC25A (Figure 6B). According to the findings of the EDU assay, the upregulation of CDC25A in CRC cells markedly reversed the inhibitory effect of cynaroside on DNA replication, and the inhibitory effect of cynaroside on DNA replication was weakened after silencing CDC25A (Figure 7A, Supplemental Figure S1A). Based on flow cytometry, the overexpression of CDC25A effectively attenuated the inhibitory effect of cynaroside on the G1/S cycle of CRC cells, and silencing CDC25A attenuated the inhibition of cynaroside in the G1/S phase (Figure 7B, Supplemental Figure S1B). The results of the Western blot analysis indicated that there was no significant alteration in the expression levels of CDC25A, CDK4, CDK6, and CDK2 in CRC cells after treatment with cynaroside and CDC25A silencing (Supplementary Materials Figure S1C). These findings suggest that the downregulation of CDC25A is associated with the mechanism by which cynaroside inhibits CRC cell proliferation.

## 3. Discussion

CRC is a malignancy that progresses to the gastrointestinal tract. CRC has a high global incidence rate and high fatality rate [17] and is the cause of approximately 600,000 deaths each year [18]. Currently, CRC treatment primarily involves systematic treatment methods, such as surgery, radiotherapy, chemotherapy, and molecular targeted drugs [19]. Although these methods can prolong patient survival, they are associated with many toxic and adverse effects and a poor prognosis [20]. Therefore, an in-depth exploration of the pathogenesis of CRC and further searches for low-toxicity and high-efficiency anti-CRC drugs remain the focus of current research [21,22].

Over the past few years, TCM has attracted the attention of many researchers worldwide owing to its unique advantages, including multiple pathways, multiple targets, and low toxicity and side effects [23]. Cynaroside is a flavonoid found in several medicinal plants and has been demonstrated to have a significant anti-tumor effect [24]. Banaszczak et al. revealed that cynaroside exerts anticancer effects by inhibiting the expression of the inflammatory factor, NF-κB, and α-amylase activity in the nucleus [25]. Velmurugan et al. demonstrated that cynaroside inhibited the migratory and invasive potential of oral cancer cells by regulating MMP-2 expression and inhibiting the expression and activity of kinases related to extracellular signal transduction [26]. Other studies have revealed that cynaroside exerts anticancer effects in non-small-cell lung cancer by triggering G0/G1 cycle arrest and autophagy [27]. More and more studies have revealed that a variety of traditional Chinese medicines have anti-tumor roles by inducing cell cycle arrest, so as to improve the quality of life and prolong the survival time of patients with advanced tumors [28,29]. In this study, cynaroside was found to promote the G1/S-phase arrest of CRC cells and suppress CRC cell proliferation. Further transcriptome sequencing revealed that CDC25A expression was considerably reduced in CRC cells after cynaroside treatment. Abnormal cell cycle regulation may be caused by cancer-related mutations, the overexpression of cell cycle regulatory proteins such as RB and CDC25, and the loss of expression of cyclin-dependent kinase inhibitors. The regulation of cell cycle plays a key role in the smooth progress of mitosis, so the research and development of anti-tumor drugs targeting cell cycles is of great significance [30,31].

CDC25A is a bi-specific protein phosphatase consisting of 524 amino acid residues, with a C-terminal catalytic domain and an N-terminal regulatory domain [32]. CDK is positively regulated by CDC25A by dephosphorylating the complex, cyclin–CDK, and activating it, thereby increasing the number of CDK cells and promoting cell cycle progression [13,33]. The upregulation of CDC25A can promote G1/S- and G2/M-phase transitions, resulting in abnormal cell cycle regulation, leading to the occurrence of tumors [34]. In this study, the level of CDC25A in CRC cells decreased after cynaroside treatment in vitro. In contrast to the expression of CDC25A in normal tissues, the upregulated expression of CDC25A in CRC tissues implies shorter overall survival. The overexpression of CDC25A significantly inhibited the effects of cynaroside on proliferation and the G1/S-phase transition in CRC cells, and the silencing of CDC25A attenuated the inhibitory effect of cynaroside on the proliferation and G1/S cycle of CRC cells.

In conclusion, cynaroside may inhibit G1/S-phase arrest and the proliferation of CRC cells by regulating CDC25A, and is thus expected to become a treatment option for CRC.

## 4. Materials and Methods

### 4.1. Cell Culture

The human CRC cell lines, HCT116 (CCL-247), HCT15 (CCL-225), RKO (CCL-2577), and LoVo (CCL-229), and the human colonic epithelial cell HCoEpiC were procured from the American Type Culture Collection. These cell lines were subsequently grown in RPMI-1640 (ThermoFisher Scientific, Waltham, MA, USA) containing 10% fetal bovine serum (FBS, Invitrogen, Carlsbad, CA, USA) and 1% penicillin/streptomycin (ThermoFisher, Waltham, MA, USA). All cells were grown in an environment containing 5% CO_2_ at a temperature of 37 °C. Cynaroside was obtained from MCE (HY-N0540, Wuhan, China) and was dissolved in DMSO. CDC25A small interfering RNA was purchased from Shenggong (Shanghai, China). The overexpression plasmid of CDC25A was purchased from GeneCopoeia (Guangzhou, China). The CRC cells were seeded in 6-well plates, and the transient transfection of overexpressed plasmids was conducted utilizing Lipo2000 (Invitrogen, Carlsbad, CA, USA) following the guidelines stipulated by the manufacturer.

### 4.2. qRT-PCR

RNA was extracted from cells using TRIzol, and a UV spectrophotometer was employed to evaluate the purity of the RNA and its concentration; this was followed by the reverse transcription of the isolated RNA into cDNA using the Super Script III First-Strand Synthesis System (Yeasen, Shanghai, China). To determine the relative number of target genes, a comparison with the internal control, β-actin, was performed. A list of the primer sequences were showed in Table 1 in this study.

### 4.3. Cell Count Kit-8 (CCK-8) Assay

A total of 3000 CRC cells were seeded in each 96-well plate. After cells adhered to the surface, they were transfected. Once the cells attached to the surface, they were placed in an incubator and exposed to various concentrations of cynaroside (0, 12.5, 25, and 50 μM). The growth medium was removed and replaced with 10 μL of CCK-8 solution (Invitrogen, Carlsbad, CA, USA) to determine cell viability. A spectrophotometer was used to determine the optical density (OD) values at 450 nm for two hours following incubation at 37 °C with the CCK-8 solution.

### 4.4. Colony Formation Assay

For the cell suspension, the stable cells were placed in 6-well plates and replenished to 2 mL at a density of 600 cells/100 μL, with each well receiving 100 μL of suspension. After 5 days of continuous culture, the cells were treated with 0, 25, and 50 μM of cynaroside. RPMI-1640 with an equal volume of DMSO was used as the control. On day 10, the medium was removed, and the cells were fixed with 4% paraformaldehyde solution for 30 min. A 0.1% crystal violet solution was used to stain the cells for 15 min. 

### 4.5. 5-Ethynyl-2′-deoxyuridine (Edu) Assay

CRC cells were grown in confocal dishes and treated with varying doses of cynaroside, ranging from 0 to 50 μM. After 24 h, the cells were incubated for 2 h in EdU medium and then treated with 0.2% Triton x-100 (Servicebio, Wuhan, China) for 10 min, followed by Apollo^®^ (RiboBio, Guangzhou, China) staining for 25 min and DAPI staining for 10 min. Finally, images were captured using a fluorescence microscope.

### 4.6. Flow Cytometry Analysis

CRC cells were cultured on confocal dishes and treated with various doses of cynaroside (0–50 μM). After 24 h, the cells were fixed with 70% ethanol overnight at −20 °C. After three separate rinses using PBS, the cells were stained with propyl iodide reagent and detected using a DeFLEX flow cytometer (Beckman, CA, USA). The results were analyzed using NovoExpress 1.2.5 software. 

### 4.7. In Vivo Assay

The animal experiments were approved by the Animal Ethics Committee of Guizhou Medical University. Nude female BALB/c mice were obtained from the Experimental Animal Center of Guizhou Medical University. Their ages ranged from 4 to 6 weeks. Mice were housed in a designated pathogen-free atmosphere under a light and dark cycle of 12 h. HCT116 cells were resuspended at a density of 2 × 10^6^ cells/mL before 100 μL of the cell suspension was subcutaneously injected into the right side of each mouse. On day 7, tumor size was measured, and mice with a size between 50 and 70 mm^3^ were selected for further study. Each mouse received an intraperitoneal injection of DMSO or 25 or 50 mg/kg cynaroside every 3 days. Tumor size was computed using (Length × Width^2^)/2. After 18 days of treatment, the mice were killed, and their kidney, liver, and tumor tissues were collected for further studies.

### 4.8. Western Blot

Total protein was extracted and added to a precooled potent lysate containing 2% PMSF protease inhibitor. After cell lysis on ice for 15 min, the cells were centrifuged at 12,000 rpm for 15 min. The protein supernatant was absorbed and quantified using the BCA method and a sample volume of 30 μg/10 μL. Protein samples (30 µg) were separated by electrophoresis on 12% separation gel at a constant voltage of 80 volts for 30 min. Electrophoresis was continued at 120 V for 1.5 h and 300 mA for 2 h. The proteins were transferred to the PVDF membrane. Milk (5%) was prepared using fresh TBST solution and sealed at ambient temperature for 2 h. The PVDF membrane was cut and labeled according to the experimental target strip. Primary CDC25A (55031-1-AP, Proteintech, Wuhan, China), CDK4 (110261-AP, Proteintech, China), CDK2 (60186-1-IG, Proteintech, China), CDK6 (4052-1-AP, Proteintech, China), and β-actin (AC038, Abclonal, Wuhan, China) at a dilution of 1:1000 were added and incubated overnight at 4 °C. The membrane strips were washed three times with TBST solution on day 2 and incubated on a shaker at room temperature for two hours. Thereafter, the secondary antibody (ratio of 1:5000) was added after each round of incubation for 20 min. The membrane strips were washed three times with TBST for 15 min each. The ultra-sensitive ECL exposure solution was used to expose the strips in a multifunction imager, and the gray values of the strips were measured using the built-in software of the imager. The relative expression levels of the corresponding proteins were calculated.

### 4.9. Hematoxylin–Eosin Staining and Immunohistochemistry

Tissue samples from the liver, kidney, and tumor were fixed in 4% paraformaldehyde before being embedded in paraffin. Hematoxylin and eosin (H&E) was used to stain 4 micron thick tissue slices of the liver and lungs. Immunohistochemistry (IHC) was performed using the tumor tissues. After osmosis, dehydration, and antigen recovery, the tumor sections were blocked with 0.3% H_2_O_2_ and 5% BSA. Thereafter, the sections were incubated with KI67 (1:200; sc-23900, SANTA, CA, USA) and PCNA (1:200; 10205-2-AP, Proteintech, China) at 4 °C for 12 h. Once the sections were rinsed thrice with PBS, they were stained with horseradish peroxidase coupled with di antibody. The immunosignals were then detected via 3,3’-diaminobenzidine staining. An optical orthophoto microscope (magnification ×200, Olympus, Tokyo, Japan) was used to collect the tumor slice fields.

### 4.10. Statistical Analysis

The results were analyzed using SPSS (version 19.0). Student’s *t*-test was used to evaluate the differences between two groups, whereas differences across multiple groups were evaluated using one-way ANOVA. The critical value of significance was set at *p* < 0.05.

## Figures and Tables

**Table 1:** The primers used in the article.

GENE	Sequence (5’ -> 3’)	
*PCNA*	Forward Primer	CCTGCTGGGATATTAGCTCCA
	Reverse Primer	CAGCGGTAGGTGTCGAAGC
*CDK4*	Forward Primer	ATGGCTACCTCTCGATATGAGC
	Reverse Primer	CATTGGGGACTCTCACACTCT
*MCM2*	Forward Primer	ATGGCGGAATCATCGGAATCC
	Reverse Primer	GGTGAGGGCATCAGTACGC
*CDC45*	Forward Primer	TTCGTGTCCGATTTCCGCAAA
	Reverse Primer	TGGAACCAGCGGTATATTGCAC
*CDC25A*	Forward Primer	GTGAAGGCGCTATTTGGCG
	Reverse Primer	TGGTTGCTCATAATCACTGCC
*E2F1*	Forward Primer	ACGCTATGAGACCTCACTGAA
	Reverse Primer	TCCTGGGTCAACCCCTCAAG
*CDC7*	Forward Primer	GAGGCGTCTTTGGGGATTCAG
	Reverse Primer	GGTCCTACTTGTAACTGTGCTG
*PLK1*	Forward Primer	AAAGAGATCCCGGAGGTCCTA
	Reverse Primer	GGCTGCGGTGAATGGATATTTC
*β-actin*	Forward Primer	CATGTACGTTGCTATCCAGGC
	Reverse Primer	CTCCTTAATGTCACGCACGAT

## Data Availability

The datasets used and/or analyzed during the current study are available from the corresponding author on reasonable request.

## Supplementary Materials

The following supporting information can be downloaded at: https://www.mdpi.com/article/10.3390/molecules29071508/s1, Figure S1. Knockdown of CDC25A recedes the G1/S-phase blocking effect of cynaroside on CRC cells. CRC cells were treated with CDC25A small interfering RNA+ DMSO and CDC25A small interfering RNA + cynaroside. (A) EDU assays were performed to determine the DNA replication rate of CRC cells in each group. (B) Flow cytometry was performed to determine the distribution of CRC cells in each group. (C) Western blot was used to detect the expression of CDC25A, CDK4, CDK6, and CDK2 in each group of CRC cells. *n* = 3. The control group was used for comparison. Data are shown as mean ± SD.

## Abbreviations

CRC	Colorectal cancer
DEGs	Differentially expressed genes
CDC25A	Cell division cycle
TCM	Traditional Chinese medicine
FBS	Fetal bovine serum
BCA	Bicinchoninic acid
CDK4	Cyclin-dependent kinase 4
