# Supplementary material for: Cynaroside Induces G1 Cell Cycle Arrest by Downregulating Cell Division Cycle 25A in Colorectal Cancer
Source: Molecules. 2024 Mar 28;29(7):1508. doi: 10.3390/molecules29071508 (PMC11013184; doi:10.3390/molecules29071508)
Supplement: Supplementary file 1 [file molecules-29-01508-s001.zip › molecules-2822664-supplementary.pdf]

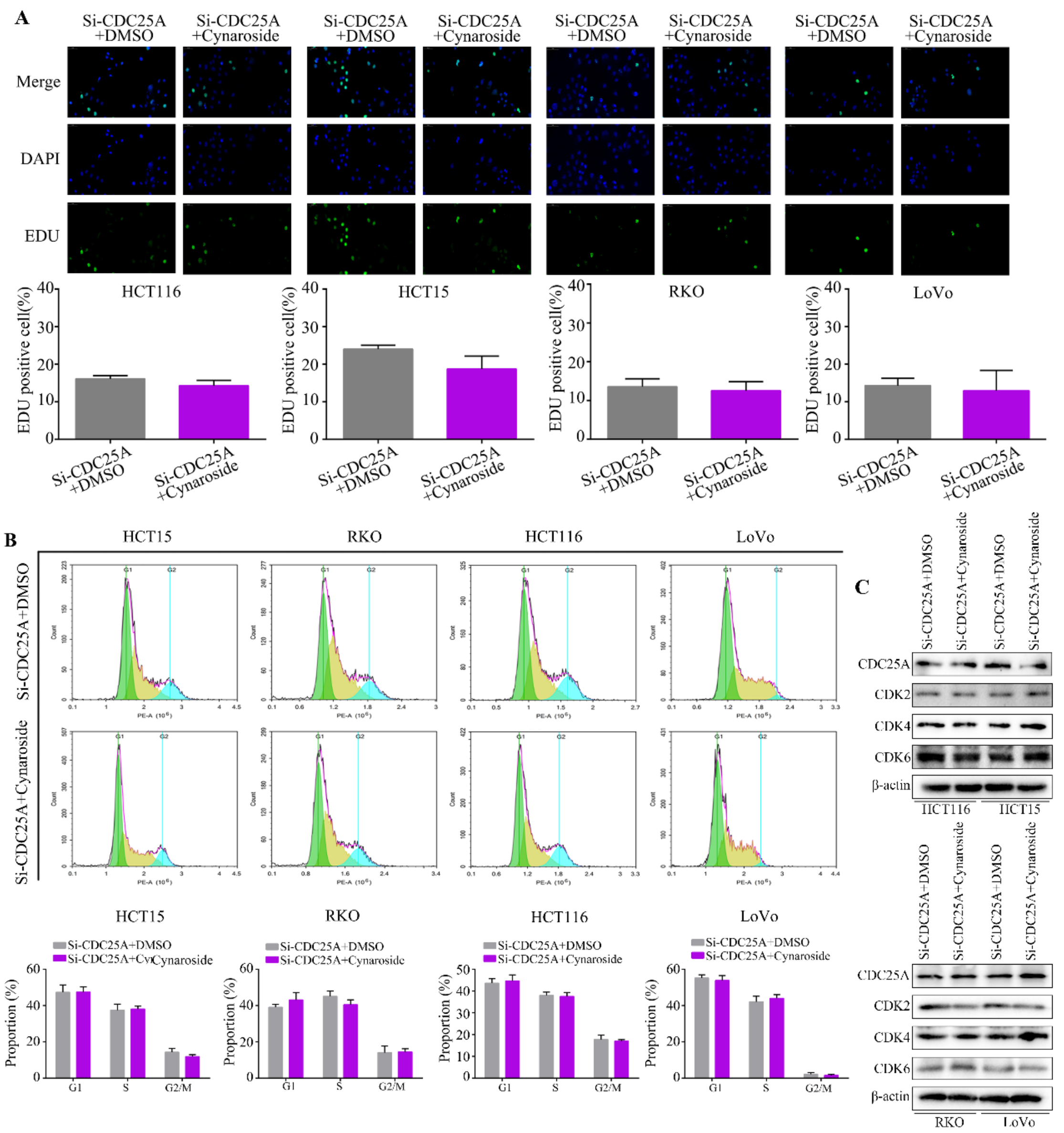

Figure S1. Knockdown of CDC25A recedes the G1/S-phase blocking effect of cynaroside on CRC cells. CRC cells were treated with CDC25A small interfering RNA+ DMSO and CDC25A small interfering RNA + cynaroside. (**A**) EDU assays were performed to determine the DNA replication rate of CRC cells in each group. (**B**) Flow cytometry was performed to determine the distribution of CRC cells in each group. (**C**) Western blot was used to detect the expression of CDC25A, CDK4, CDK6, and CDK2 in each group of CRC cells. n = 3. The control group was used for comparison. Data are shown as mean ± SD.
